# Structural, Electrical, and Mechanical Properties Investigation of Open-Cell Aluminum Foams Obtained by Spark Plasma Sintering and Replication on Polyurethane Template

**DOI:** 10.3390/ma15030931

**Published:** 2022-01-26

**Authors:** Alexandra Kosenko, Konstantin Pushnitsa, Artem Kim, Pavel Novikov, Anatoliy A. Popovich

**Affiliations:** Institute of Machinery, Materials, and Transport, Peter the Great Saint Petersburg Polytechnic University, Politechnicheskaya ul. 29, 195251 Saint Petersburg, Russia; pushnitsa.k@gmail.com (K.P.); artem_7.kim@mail.ru (A.K.); novikov.p.a@gmail.com (P.N.); popovicha@mail.ru (A.A.P.)

**Keywords:** open-cell foams, porous materials, spark plasma sintering, replication technique, PU foam template

## Abstract

The present paper illustrates a comparison of open-cell aluminum foams. The foams were fabricated by two different methods: spark plasma sintering and replication on a polyurethane template. The influence of pressure, temperature, and diameter of space holding material on foam obtained by the spark plasma sintering method was investigated. Additionally, the aluminum powder content in slurry and atmosphere during thermal processing of foam prepared by the replication technique were studied. The morphology and structure of obtained samples were analyzed by scanning electron microscopy and X-ray diffraction analysis. Supplementarily, mechanical properties and electrical conductivity were studied. The porosity of obtained samples was 83% for the SPS sample and 85% for the replication sample. The results of the studies carried out gave us an understanding that the SPS method is more promising for using the obtained foams as cathode current collectors in lithium-ion batteries due to excessive aluminum oxidation during sintering in the furnace.

## 1. Introduction

Metal foams are currently a special class of lightweight materials that can be used in various fields of the industry due to their physical, chemical, and mechanical properties [1,2,3]. Using metal foam instead of solid metal is more efficient and reduces the weight and cost of the final product [2]. Metal foam might be able to substitute a variety of conventional materials. Currently it is used in a wide range of engineering applications. Therefore, this fact allows applying these materials in various fields such as automobiles, aerospace, and naval industries [3]. It is possible to manufacture metal foam from pure metals, for example, titanium, nickel, and aluminum [4,5], and in the same way from different alloys [6,7]. Due to the lightweight nature of this type of material, metal foam is an extremely promising material for the automotive industry; in particular, open-cell foams made of aluminum and Al alloys are of special interest. Remarkable properties of open-cell aluminum foam such as low density, high specific electrical and thermal conductivity, and high surface area allow to be applied as a component of the lithium-ion battery, specifically to use aluminum foam instead of aluminum foil as cathode current collectors [8,9,10,11].

A variety of fabrication processes have been developed to produce metal foams, in particular aluminum foams [12,13,14,15,16,17,18,19,20]. The common processes are either via the addition of a foaming agent into molten aluminum alloy or through bubbling gas into a molten aluminum alloy. The main drawbacks are that aluminum foams produced by these methods are limited in terms of control of the pore content and size and microstructure of cell walls. The methods of production for open-cell aluminum foam can be divided into three classes: solid-state methods, liquid metallurgy methods, and vapor state methods [21]. Solid-state methods include the sintering of metal powder. It is well known that spark plasma sintering (SPS) decreases the sintering time and temperature, compared with sintering in an electric furnace, resulting in suppression of grain growth during sintering [22,23,24,25]. Therefore, it is worthwhile to investigate the microstructure and mechanical properties of aluminum foam processed by SPS. In addition, one of the promising ways to manufacture open-cell metal foams is the sponge replication technique, and this method has been successfully applied for the production of open-cell titanium, copper, and aluminum foams [26,27,28,29,30,31,32,33]. The main disadvantage of the replication method is the high oxidation rates of powders during the sintering step [34,35].

In the present paper, open-cell aluminum foams, fabricated by spark plasma sintering and by sponge replication technique, are investigated. The necessity of the research performed was in changing the standard current collector in lithium-ion batteries, aluminum foil, to aluminum foam, due to aluminum foam being a more lightweight and effective type of material. The main difference between this research and previously published ones is that no additives, such as foaming and thickener agents, were used. This fact can lead to a low impurity content in composition of the resulting foams, which is important in the further use of the foam. The investigation focused on foam production with a highly open structure, with low impurity content, a lower amount of oxide film on the surface, high electrical conductivity, and sufficient mechanical strength. The last two points are high priority in this research. Mechanical strength is necessary since the aluminum foam must not collapse when the active cathode material is applied. High electrical conductivity is important for low internal resistance and high specific energy capacity of the battery.

## 2. Materials and Methods

### 2.1. Materials

**Figure 1:**
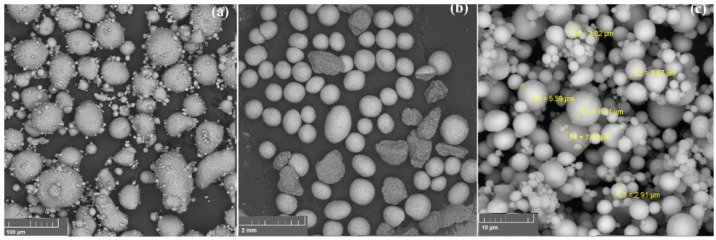
(**a**) Aluminum alloy powder, (**b**) sodium chloride, (**c**) aluminum powder.

Figure 1 represents an SEM image of the aluminum alloy powder used for SPS method (a), sodium chloride (b), and aluminum powder used for replication technique (c) obtained in the backscattered electron detection mode. Aluminum alloy powder particles are predominantly rounded, the particle size distribution is as follows: d_10_ = 20.9 μm, d_50_ = 51.6 μm, d_90_ = 95.9 μm. It means that 10% of particles have a size less than 20.9 μm, 50% of particles have a size less than 51.6 μm, and 90% of particles have a size less than 95.9 μm. Additionally, there are some submicron size particles. The powder has a homogeneous structure. In Figure 1b sodium chloride particles are presented. Particles have mostly spheroidal and round form, the powder was sieved and fraction 500–1000 μm was chosen. Figure 1c shows the aluminum powder particles, all of the particles have a spherical shape, a homogeneous structure, and the particle size distribution is d_10_ = 2.4 μm, d_50_ = 5.5 μm, d_90_ = 10.8 μm.

### 2.2. Spark Plasma Sintering Method

**Figure 2:**
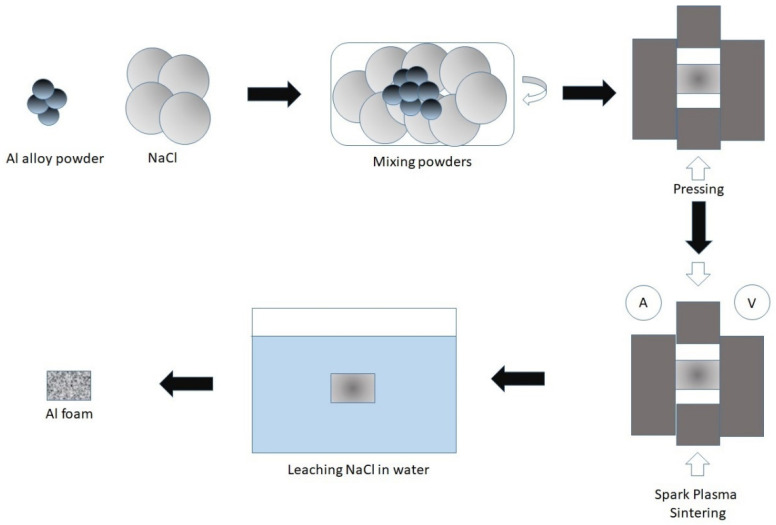
Schematic illustration of spark plasma sintering (SPS) method.

The commercially available aluminum alloy powder (AMD5, supplier Normin LTD., Oxford, UK) with the content of Al 94.8 wt.%, Mg 4.8 wt.%, Ti 0.4 wt.%, with an average particle size of 90 μm, was used as a starting material. It is well known that Al–Mg alloys are characterized by a combination of satisfactory strength, good ductility, very good weldability and corrosion resistance [36]. Titanium in Al alloys also enhances microstructural and mechanical properties, including specific strength, oxidation and corrosion resistance [37]. Sodium chloride (supplier LenReactiv JSC, Saint Petersburg, Russia) with average particle size 500–1000 μm, was used as a space holder for SPS. The weight ratio of the aluminum powder to NaCl was determined to be 10:90 and the volume ratio 8:92. To select the optimal weight ratio of aluminum powder content and porosity of the final Al foam, mixtures with a content of 5% and 10% were prepared and foams with the same sintering conditions were made. In the case of 5%, the sample retains its shape, but begins to crumple with a slight pressure with tweezers, in the case of samples of 10% this is not observed. The spark plasma sintering method is schematically presented in Figure 2. In general, the process consists of 4 steps: mixing the aluminum powder and sodium chloride, pressing, sintering, and leaching in water. Firstly, the aluminum powder and space-holding material were mixed in the vibrating mixer (C50.0 Vibrotechnik LLC, Saint Petersburg, Russia) for 90 min. After the ingredients were homogeneously mixed, the resulting powder was pressed in graphite mold at a pressure of 5 MPa. Then, sintering was carried out on the equipment for spark sintering FCT system HPD 25 with the following parameters: sintering temperature 550 °C, pressure 38 MPa, sintering time 5 min, pulse duration 2–4 ms, pause duration 2 ms. The sintered sample after the sintering was placed into hot water for 12 h to leach out the embedded sodium chloride particles, to obtain the aluminum foam with porous structure.

### 2.3. Sponge Replication Technique

**Figure 3:**
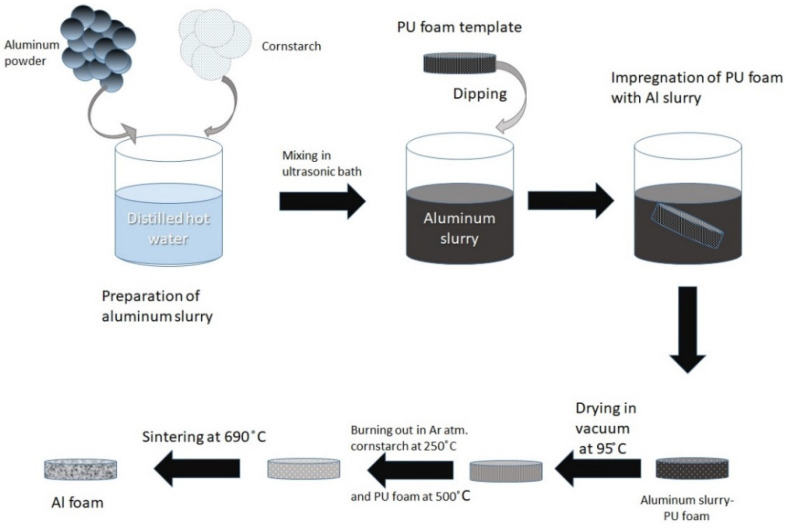
Schematic illustration of replication technique.

The commercially available aluminum powder with purity 99% and with particle size <10 μm was used (ASD6, supplier Normin LTD). The aluminum powder was mixed with earlier-prepared 0.4 wt.% cornstarch solution in distilled water. Cornstarch is a binder in this solution. The content of Al powder in final dispersion was 80 wt.%. The dispersion was mixed by using an ultrasonic bath to make it homogeneous. This method consists of 3 steps: (1) coating a polyurethane (PU) sponge with a powder slurry; (2) burning the PU and binder out; (3) final sintering of the foam. The schematic illustration is below in Figure 3. An open-cell polyurethane foam cylinder (Polinazell PPI 20. supplier United Service Company LLC, Chicago, IL, USA) with a height of 3 mm and a diameter of 19 mm was used as a template.

As a first step, the PU foam was drowned into previously mentioned dispersion, soaked for 5 min, and then the excess was removed by mechanical wringing. After this step, the dispersion-coated template was dried for 17 h in a vacuum at a temperature of 95 °C. Further, the sample was placed in a tube electric furnace to burn out the PU foam and binder. The final thermal processing step was carried out also in a tube electric furnace. All burning and thermal processes were conducted in the Argon atmosphere, the flow rate was 40 mL/min. The conditions of processing are listed below in Table 1. Due to the cooling effect of Argon flow, the sintering temperature is higher than the melting point of aluminum.

### 2.4. Characterisation

The morphology, microstructure, and chemical composition of the samples were studied using Mira 3 Tescan scanning electron microscope with an EDX Oxford Instruments X-max 80 energy dispersive detector for X-ray spectroscopy. X-ray phase analysis was carried out on a Bruker Advance D8 diffractometer in the range of angles from 25° to 85° with a step of 0.02° and an exposure of 1.5 s at each step. The wavelength was 1.5406 Å. The lattice parameters were measured by the Le Bail method using software TOPASS. The particle size (PS) distribution of the powders was studied using a Fritsch Analysette 22 NanoTec plus laser diffraction unit. To calculate the particle size distribution, the Fraunhofer model was used. The electrical conductivity was measured using an AKIP-2101 voltmeter, a GPD-74303S current source, and a FLUKE-289 multimeter. Mechanical compression tests of the specimens were carried out on a Zwick/Roell Z100 testing machine.

## 3. Results and Discussion

### 3.1. Aluminum Foam Formation

In Table 2 different conditions of sintering are demonstrated. First, it is worth noting the fact that at a temperature of 600 °C and a pressure of 19 MPa and above, aluminum powder melts and flows out of the mold. At 500 °C, and pressure 38 MPa and below, spherical non-deformed particles are observed, which indicates worse sintering. however, at pressure above 38 MPa the powder starts to melt. The best results of sintering were obtained at temperature 550 °C and pressure 38 MPa.

**Figure 4:**
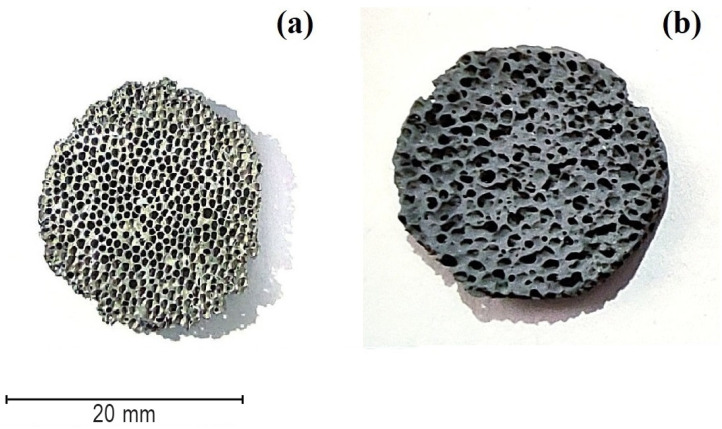
Obtained open-cell aluminum foam by SPS (**a**), by replication method (**b**).

In Figure 4 the obtained samples of aluminum foam are presented. Figure 4a is the SPS sample, and Figure 4b is the replication sample. At first glance, it seems that the SPS sample has more open porosity than the sample fabricated by replication technique; in the next subsections the physicochemical and mechanical properties are considered in more detail. The shape and size of the samples were selected on the basis of further research as current collectors in lithium-ion batteries. The diameter and height of the samples should be no more than 20 mm and 2 mm, respectively, so that they can be placed in the CR2032 case for further testing.

### 3.2. Morphology and Microstructure Analysis

**Figure 5:**
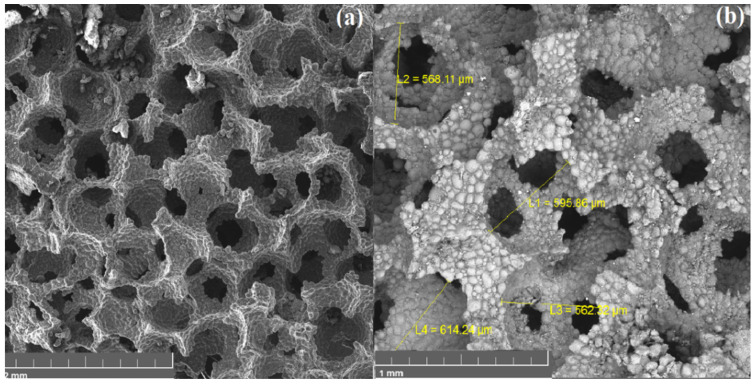
SEM image of foam obtained by SPS method: (**a**) general view, (**b**) pore size investigation.

Figure 5 shows the surface morphology of a sample obtained by the method of spark plasma sintering. The sample has a cellular structure with open porosity. It is apparent that the material has a homogeneous structure with a pore diameter of about 600 μm. It is possible to control the pore diameter of fabricating foam by choosing different fractions of space holding material—sodium chloride.

**Figure 6:**
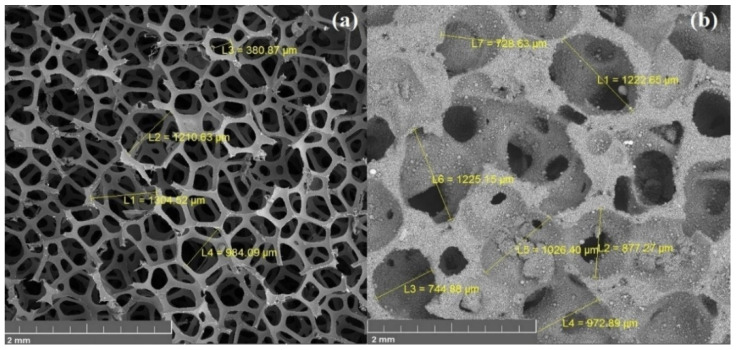
SEM image of PU sponge (**a**) and obtained foam by replication technique (**b**).

Figure 6 shows images of the original polyurethane sponge and a sample of aluminum foam obtained by the replication method. The sample has open porosity. The pore diameter is in a wide range (300–1000 microns) and it is comparable to the pore size of the PU sponge. Small pores can be seen on the replication sample due to the incomplete repetition of the template relief, which is justified due to the high viscosity of the suspension.

**Figure 7:**
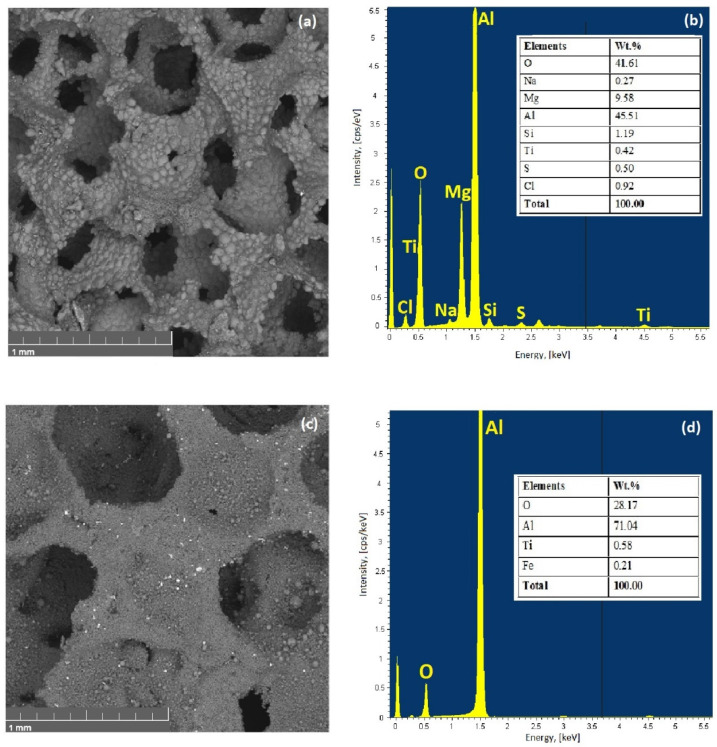
SEM image (**a**,**c**) and corresponding energy-dispersive X-ray spectroscopy spectra (**b**,**d**) of SPS sample (**a**,**b**) and replication sample (**c**,**d**).

From EDX spectra (Figure 7b) it can be concluded that spreading of alloy elements in the SPS sample after sintering is uniform, and the amount of sodium and chlorine is less than 1 wt.%; the presence of sulfur is due to its impurity in the composition of the salt. The results of the replication sample EDX analysis of Figure 7d represents that the chemical composition after thermal processing is characterized by 70.8 wt.% aluminum, and a large amount of the oxygen is recorded as well. However, this method gives a large error when measuring the number of light elements.

**Figure 8:**
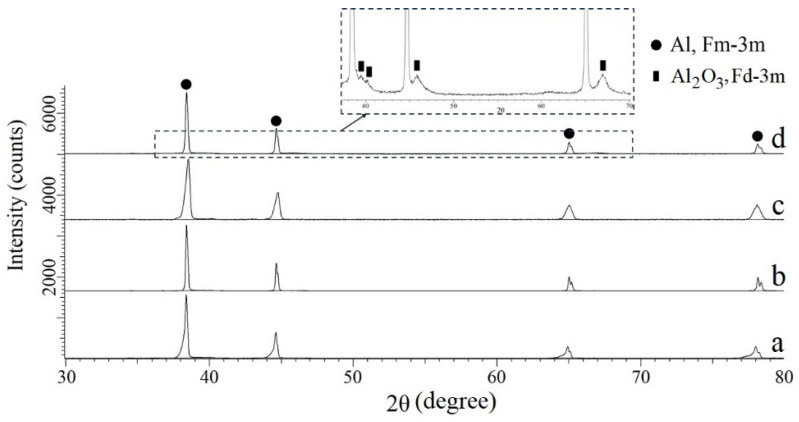
Diffraction patterns of Al alloy powder (**a**), Al powder (**b**), SPS sample (**c**), and replication sample (**d**).

Figure 8 shows the diffraction patterns of Al alloy powder (a), Al powder (b), the sample obtained by the SPS (c), and by the replication method (d). The main peaks of all diffraction patterns are indexed by the cubic Al phase (PDF 03-065-2869) with the space group Fm-3m. The unit cell parameters obtained by the Rietveld method are presented in Table 3. In the sample obtained by the replication method, the presence of the γ-Al_2_O_3_ phase is observed.

### 3.3. Porosity

The density of the aluminum foams was determined by hydrostatic weighing (Archimedes method). As a first step, the samples were weighed in air, then they were coated with paraffin to prevent the fluid from entering inside the foam pores. After this step, the samples were weighed in air and further in ethanol, the density of which amounts to 0.785 g/cm^3^ at 25 °C. The relationship between the porosity *P_r_* (%) of the aluminum foam and the relative density (*ρ_f_*/*ρ_s_*) of the foam are presented in Equation (1), where *ρ_f_* and *ρ_b_* represent densities of the foam sample and bulk aluminum (about 2.7 g/cm^3^), respectively. The density of the aluminum foam was determined by hydrostatic weighing. The measurements of samples densities and porosity are presented in Table 4.

(1) $$P_{r}=\left(1-\frac{\rho_{f}}{\rho_{b}}\right)\times 100$$

### 3.4. Electrical Conductivity

The electrical conductivity of samples was determined by measuring the resistance using the 4-wire testing method. Electrodes for measuring the potential difference were installed directly on the sample with a constant distance between electrodes (10 mm) for each measurement. The accuracy of measuring the potential difference with the used device was 0.01%.

To determine the resistance of the samples, a direct current was applied. The accuracy of the applied current was ±0.5%. To clarify the measured results, the current was measured with an ammeter with an error of no more than ±0.2%. The potential of aluminum foil and samples was measured. Aluminum foil is a frequently used material as a cathode current collector in lithium-ion batteries. In this test, it was used as a reference to compare with. The diameter of the foil was 20 mm, the thickness, 14 μm. For each sample, three measurements were taken at different locations. The resistance of the samples for each measurement was calculated using Ohm’s law equation. The results of the measurements are presented in Table 5.

The key factors affecting the electrical conductivity of the samples are the degree of sintering and the amount of aluminum oxide on the surface of the samples. The determination of the electrical conductivity of the samples showed results consistent with the X-ray analysis—the sample obtained by replication technique has the worst electrical conductivity, which can be explained by the formation of aluminum oxide film on the sample surface and the surface of grains. In addition, the calculated resistances of aluminum foil and SPS sample gave us an understanding that the SPS sample is the closest in electrical conductivity to aluminum foil. The obtained results demonstrate that the SPS sample obtained due to its properties is better for application as a three-dimensional current collector than the sample obtained by replication.

### 3.5. Mechanical Properties

**Figure 9:**
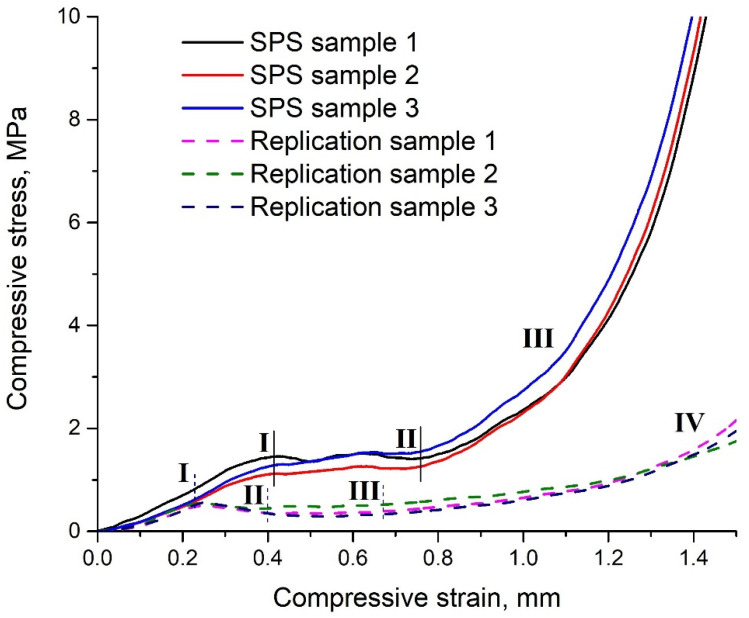
Compression strain curves for aluminum foams: SPS sample, replication sample.

The compression stress–strain curves of the obtained samples are shown in Figure 9. The dimensions of samples were: diameter—19 mm, height—2 mm. Three samples of each obtaining method were taken for the compressive test. For both samples, coefficient of variation was calculated. The average coefficient of variation for SPS samples was 12%, and for replication samples was 13%.

The stress–strain curves of the SPS sample are characterized by three distinct regions (designate by solid lines). The first region is an elastic region characterized by a linear elastic region at very low deformation without peak stress. The second area is the plateau area on which pores collapse (destruction of the bridges), which may indicate the growing deformation without increasing pressure. Fluctuations of the curve in this area can be associated with the uneven distribution of pores in the sample. The third area is the area of compaction, where a sharp increase in compressive stress occurs.

The stress-strain curve of the replication sample is characterized by four regions (designate by dash lines). The first region is the region of elastic deformation; relative to the SPS sample, the region of elastic deformation ends at a lower pressure. In the second region, the pressure decreases with the continued growth of deformation, which may indicate not plastic compression of the bridges, but their brittle fracture. This behavior is demonstrated by all samples. The brittle fracture occurs due to the presence of aluminum oxide in the sample. The third region is less pronounced than for the SPS sample due to the smaller amount of aluminum in the sample. In the fourth region, the same process occurs as in region three for the SPS sample.

For the samples, the adsorbed energies were also compared during mechanical strength tests. Due to the small thickness of the sample, the tests were carried out without using an extensometer, which would not allow the calculation of the nominal adsorbed energy. However, it is admissible to compare the adsorbed energies of the two samples. The calculation of the adsorbed energies was carried out according to the method presented in the article [38]. The ratio of the adsorbed energies of the samples (a)/(b) was 5.24. This value shows that, upon compression, sample (a) absorbed 5.24 times more energy, which also indirectly indicates the metallic structure of sample (a) and the nonmetallic structure of sample (b).

### 3.6. Properties Comparison of Samples

Babcsán et al. have used the foam in their studies, manufactured by the batch casting process. It is possible to obtain aluminum foam with closed porosity by this method. The main difference between our method and theirs is that we pursued the goal of obtaining open-cell aluminum foam. Additionally, our research is distinguished from the Babcsán research since no additives were used in the foam production. In their method, calcium and titanium hydride were used as thickener and foaming agent, respectively. Using our method, as a result, we obtained a sample with a minimum content of impurities. Impurities, which were found by EDX analysis, also were present in the initial materials.

The comparison of samples is presented in Table 6. The low conductivity of the SPS sample can be explained by incomplete sintering of powder during the formation of the sample. The replication sample result was previously explained: the large amount of aluminum oxide has influence on the conductivity.

## 4. Conclusions

Open-cell aluminum foams were successfully obtained by two various methods: spark plasma sintering and replication on polyurethane template. The porosity of final samples was 83% and 85%, respectively. According to the investigations carried out on the microstructure and morphology, electrical conductivity, and mechanical properties of obtained samples, we can conclude that the sample obtained by the replication method has more aluminum oxide film on its surface than the SPS sample. The consequence of this fact is the lower electric conductivity of the replication sample. The electrical conductivity of the SPS sample is comparable to the aluminum foil. This fact allows us to assume that the electrochemical behavior of the SPS sample will be similar to the foil as a part of lithium-ion batteries. Mechanical tests of the foams also demonstrated the presence of a brittle phase in the replication sample. The SPS sample showed metallic properties and determined sufficient mechanical strength. Due to investigated properties of the SPS sample, it is evident that the spark plasma sintering method is the most promising way to produce open-cell aluminum foam for further use in lithium-ion batteries.

The future research plan is to find the optimal balance of temperature, pressure and sintering time in order to improve the processing mode of the SPS method. Additionally, the further improvement of the samples’ characteristics is possible by using fluxes, which limit the oxide film growth and improve the sintering of the aluminum particles. The next step is to assemble mock-ups of CR2032 batteries to study the influence of a new type of current collector on batteries’ behavior using cyclic voltammetry and electrochemical impedance spectroscopy methods.

## Figures and Tables

**Table 1:** Conditions of binder and PU burning and sintering of aluminum foam sample.

Conditions	Binder Burning	PU Burning	Sintering
Initial temperature, °C	20	470	520
Final temperature, °C	470	520	690
Heating time, min	60	25	20
Holding time, min	0	0	480

**Table 2:** Different conditions of spark plasma sintering method.

Pressure/ Temperature	19 MPa	29 MPa	38 MPa	48 MPa	57 MPa
500 °C	Not sintered	Not sintered	Not sintered	Melted	Melted
550 °C	Not sintered	Partly sintered	Sintered sample	Melted	Melted
600 °C	Melted	Melted	Melted	Melted	Melted

**Table 3:** Unit cell parameters were obtained by the Le Bail method.

Sample	Al Alloy Powder	Al Powder	SPS	Replication
***a*, Å**	4.062	4.049	4.062	4.051

**Table 4:** The measurements of density and porosity of aluminum foam.

Sample	Dry Weight, g	Dry Weight with Paraffin, g	Underwater Weight, g	Density, g/cm^3^	Porosity%
SPS	0.278	0.621	0.125	0.45	83%
Replication	0.328	0.783	0.164	0.42	85%

**Table 5:** The resistance measurement of samples by using the 4-wire testing method.

Sample (Measurement)	Applied Current, A	Measured Potential Difference, V	Calculated Resistance, Ohm
Aluminum foil 1	0.149	0.002	0.013
Aluminum foil 2	0.151	0.002	0.013
Aluminum foil 3	0.148	0.001	0.007
SPS 1	0.153	0.001	0.006
SPS 2	0.157	0.002	0.012
SPS 3	0.156	0.001	0.006
Replication 1	0.151	47.291	313.185
Replication 2	0.155	49.517	319.464
Replication 3	0.148	46.536	314.432

**Table 6:** Properties comparison of open-cell aluminum foams.

Property	Babcsán, N et al. Sample [39]	SPS Sample	Replication Sample
Porosity, %	83	83	85
Density, g/m^3^	0.46	0.45	0.42
Electrical conductivity, S/m	3.43 × 10^6^	1.39 × 10^4^	3.18 × 10^−1^

## Data Availability

The data presented in this study are available on request from the corresponding author.
